# Renal Artery Stenosis and Its Predictors in Hypertensive Patients Undergoing Coronary Artery Angiography

**DOI:** 10.5812/iranjradiol.4553

**Published:** 2011-12-25

**Authors:** Hakimeh Vahedparast, Mohammad Reza Pourbehi, Abdullatif Amini, Maryam Ravanipour, Shokrollah Farrokhi, Kamran Mirzaei, Nima Nasehi

**Affiliations:** 1 Faculty of Nursing and Midwifery, Bushehr University of Medical Sciences, Bushehr, Iran; 2 Department of Cardiology, Faculty of Medicine, Bushehr University of Medical Sciences, Bushehr, Iran; 3 The Persian Gulf Tropical and Infectious Disease Research Center, Bushehr University of Medical Sciences, Bushehr, Iran; 4 Department of Immunology and Allergy, Medical College, Bushehr University of Medical Sciences, Bushehr, Iran; 5 The Persian Gulf Biomedical Research Institute, Bushehr University of Medical Sciences, Bushehr, Iran; 6 The Persian Gulf Nuclear Medicine Research Centre, Bushehr University of Medical Sciences, Bushehr, Iran; 7 Department of Community Medicine, Faculty of Medicine, Bushehr University of Medical Sciences, Bushehr, Iran; 8 Department of Radiology, Fatemeh Zahra Hospital, Bushehr University of Medical Sciences, Bushehr, Iran

**Keywords:** Renal Artery Obstruction, Coronary Artery Disease, Hypertension, Angiography

## Abstract

**Background:**

Renal artery stenosis (RAS) has been increasingly recognized in the recent years, especially in patients with coronary artery disease (CAD). RAS affects the patients with hypertension (HTN), but the exact prevalence is not known.

**Objectives:**

This study was performed to determine the prevalence and to identify the predictors of RAS in hypertensive patients undergoing coronary artery angiography.

**Patients and Methods:**

In a cross-sectional study from August 2008 to August 2009, 481 patients with HTN and suspected CAD underwent selective coronary and renal angiography for screening and predicting RAS. RAS was defined as a higher than 50% stenosis in the renal artery lumen. Multivariate analysis of factors associated with the presence of RAS were examined using a logistic regression model.

**Results:**

The mean ± standard deviation of age was 59.25 ± 10.81 years and 50.3% were men. According to angiographic data, 425 patients (88.4%) had CAD, while 56 (11.6%) had normal coronary arteries. RAS was seen in 94 (22%) patients with CAD. The multivariate logistic regression analysis identified only age (P < 0.001) and the number of significant coronary lesions (P < 0.001) as independent predictors of RAS. Gender, smoking, congestive heart failure, diabetes mellitus (DM), hyperlipidemia (HLP) and body mass index (BMI) were not independent predictors.

**Conclusions:**

This study suggests that in the management of patients with RAS, risk factors should most likely be considered as beneficial. In addition, the clinical and angiographic features are helpful in predicting its presence in elderly patients with CAD.

## 1. Background

Renal artery stenosis (RAS) is defined as thinning of the renal artery duct. The most common cause of RAS, atherosclerosis, usually involves the main renal artery and the perirenal aorta [1]. The prevalence of RAS in the general population is unknown [2]. The prevalence of RAS in patients with suspected cardiovascular disorders who have undergone cardiac catheterization was reported as 11.3 to 39%, whereas the RAS with stenosis of more than 50% has been reported as 6.3 to 28% [3]. Furthermore, RAS affects 1 to 5% of patients with blood hypertension (HTN), while it is the most common cause of secondary HTN. RAS can occur unilaterally, bilaterally, or in relation to a solitary functioning kidney [4][5]. Studies have demonstrated that coronary artery disease (CAD) is one of the most important causes of death in patients with atherosclerotic RAS. In addition, a high prevalence of RAS has been reported in patients with documented CAD [6][7]. Contrast angiography is a standard method for the detection of RAS that is readily performed in combination with coronary angiography [8]. Therefore, using abdominal aortography concomitant with coronary angiography, a close relationship between the extent of CAD and the severity of RAS has been confirmed [9][10].

Factors associated with the prevalence of RAS are reported as old age, CAD, a higher number of prescribed cardiovascular drugs, HTN, female gender and a previous coronary artery bypass graft [11]. Therefore, the early detection and management of RAS, which usually exhibits subtle clinical manifestations, is more important for patients in the presence of unexplained renal insufficiency, refractory HTN, or episodes of unexplained cardiac decompensation.

## 2. Objectives

Due to lack of information about the incidence of RAS and its related risk factors in patients with HTN in the Bushehr population, this prospective study was implemented to determine the prevalence and severity of RAS and its association with predictive variables of atherosclerosis in patients with HTN who are referred for coronary angiography.

## 3. Patients and Methods

### 3.1. Study Group

In a cross-sectional study from August 2008 to August 2009, we prospectively performed selective renal arteriographies in 835 consecutive patients admitted for possible cardiac intervention at the Department of Coronary Angiography, Bent Al-Hoda Hospital of Bushehr University of Medical Sciences. Before catheterization, a protocol-based clinical examination was used to determine the demographic data, atherogenic risk factors such as diabetes mellitus (DM), HTN, hyperlipidemia (HLP), smoking status and body mass index (BMI). Patients presenting with congenital heart disease or patients hemodynamically unstable during catheterization, known or suspected cases of acute renal failure, patients with a history of contrast nephropathy, patients who were not considered based on the physician’s preference and patients who refused or were not able to provide informed consent were excluded from this study. The study was approved by the local Ethics Committee. Written informed consent was obtained from all subjects before any intervention.

We selected patients with HTN and suspected CAD who underwent coronary angiography (n = 481 of 835). The patients subsequently underwent renal artery angiography via the femoral approach (Seimens, Germany). In this study, the cutoff point used for the definition of RAS and CAD were defined as a higher than 50% stenosis in diameter which was considered as significant. RAS was independently reported by two interventional cardiologists and assessed by visual estimation as offline. RAS was graded into two groups of normal or less than 50% stenosis and more than 50% stenosis. In case of disagreement between the angiographists the patients were excluded from the study.

### 3. 2. Statistical Analysis

Demographic and procedural data are expressed as frequency and mean ± SD. The prevalence and grade of RAS for each group were determined. A binary variable analysis of factors associated with presence of RAS was carried out using a logistic regression model (forward method). P value less than 0.05 was considered significant.

## 4. Results

Of all the 835 hypertensive patients who were candidates for coronary angiography, totally 481 (57.6%) were included in the study and 354 patients were excluded because of the exclusion criteria. The enrolled patients underwent renal angiography too. Two-hundred forty two of the patients (50.3%) were male and 239 (49.7%) were female. According to the demographic data, the patients’ risk factors were HLP (43.0%), DM (30.4%) and smoking (41.8%). Based on the definition of RAS in this study, totally 136 (28.3%) of the patients had RAS while bilateral normal renal arteries or less than 50% stenosis were identified in 345 (71.7%) of the screened patients. The mean age ± SD was 59.25 ± 10.81 and the age ranged from 33 to 88 years with a mean ± SD of 57.87 ± 10.51 in the normal and 62.73 ± 10.81 in the RAS group (P < 0.001). The mean ± SD of BMI in the normal and RAS groups was 26.83 ± 7.01 and 26.25 ± 5.49, respectively (P > 0.05) (Table 1). Significant unilateral RAS was identified in 11.1%, while 17.2% had significant bilateral RAS. Based on significant stenosis (RAS) in the right or left artery, there were 108 (23.7%) and 120 (22.5%) patients with RAS in the right or left artery, respectively (Table 2). Our findings from coronary artery angiography revealed that 11.7% had a normal coronary artery or an artery without stenosis and 10.4% had less than 50% stenosis in their coronary vessels, while 18.33% of the patients had single vessel disease (1 VD), 19.6% had two-vessel disease (2 VD), 35.4% had three-vessel disease (3 VD) and 4.2% had both 3 VD and left main coronary artery (LMCA) disease. In addition, the frequency of RAS regarding the number of the involved coronary arteries was 18 (20.5%) of the 88 patients with 1 VD, 27 (28.7%) of the 94 patients with 2 VD, 62 (36.5%) of the 170 patients with 3 VD and nine (45%) of the patients with LMCA together with 3 VD. Bivariate analysis showed that patients older than 70 years (P < 0.001) and the number of significant coronary lesions (P < 0.001) was significantly associated with the presence of RAS. RAS was not significantly correlated with DM, HLP, high BMI or smoking (P > 0.05) (Table 1).

**Table 1:** Factors Associated With Renal Artery Stenosis

	**β Coefficient**	**SE a**	**OR a**	***P* value**	**95% CI a**
Age, y					
< 60	1				
60-69	-1.258	0.273	0.284	0.004	0.0167-0.485
> 70	-0.843	0.0289	0.430	0.000	0.244-0.751
Sex					
Male	1				
Female	-0.64	0.241	0.938	0.789	0.585-1.504
DM a					
No	1				
Yes	-0.081	0.260	0.922	0.775	0.553-1.536
BMI a, kg/m^2^					
< 21	1				
21-24	-0.881	0.377	0.414	0.089	0.198-0.868
25-29	-0.644	0.368	0.525	0.08	0.255-1.08
> 30	-0.342	0.411	0.710	0.405	0.308-1.589
Smoking					
No	1				
Yes	0.187	0.238	1.205	0.433	0.756-1.589
HLP a					
No	1				
Yes	0.052	0.246	1.053	0.833	0.651-1.705
LVEF a					
> 50	1				
30-50	0.246	0.622	1.303	0.671	0.385- 4.408
< 30	-0.156	0.288	0.855	0.587	0.487- 1.503
CAD a					
Normal	1				
1 VD	0.426	0.3	0.653	0.156	0.363- 1.176
2 VD	-0.849	0.345	0.428	0.04	0.218- 0.842
3 VD	-1.067	0.361	0.344	0.003	0.170 – 0.699

^a^ Abbreviations: BMI, body mass index; CAD, coronary artery disease; CI, confidence interval; DM, diabetes mellitus; HLP, hyperlipidemia; LVEF, left ventricular ejection fraction; OR, odds ratio; SE, standard error

**Table 2:** Demographic Characteristics of Participants

	** Renal Artery Stenosis Condition**	
	**Normal or < 50% Stenosis (n = 345)**	**Renal Artery Stenosis (n = 136)**
Age, y, No. (%)		
< 60	189 (55.3)	51 (37.5)
60-69	101 (29.5)	39 (28.7)
> 70	52 (15.2)	46 (33.8)
Gender, No. (%)		
Male	174 (50.4)	68 (50)
Female	171 (49.6)	68 (50)
BMI a, kg/m^2^, No. (%)		
21 <	31 (9.3)	20 (15.6)
21-24	115 (34.5)	38 (29.7)
25-29	122 (36.6)	43 (33.6)
> 30	65 (19.5)	27 (21.1)
Diabetes Mellitus, No. (%)		
No	237 (68.7)	98 (72.1)
Yes	108 (31.3)	38 (27.9)
Smoking, No. (%)		
No	194 (56.2)	83 (63.2)
Yes	151 (43.8)	50 (36.8)
Hyperlipidemia, No. (%)		
No	191 (55.4)	83 (61)
Yes	154 (44.6)	53 (39)
Renal artery stenosis condition, No. (%)		
Right	367 (76.3)	114 (23.7)
Left	373 (77.5)	108 (22.5)
Both	344 (71.7)	136 (28.3)

^a^ Abbreviation: BMI, body mass index

## 5. Discussion

The prevalence of RAS in our study was higher than previous studies, mainly in patients with atherosclerosis in the coronary artery, cerebrovascular or peripheral vascular diseases undergoing abdominal aortography [3]. Some investigations have reported that the prevalence of RAS among patients who underwent coronary angiography was usually between 6.2% and 28% in Western countries, but there is little information about the prevalence of RAS and its predictors in the Middle East [12]. The prevalence of RAS was reported as higher than 20% in studies among selected patients such as patients who undergo diagnostic coronary angiography [13]. For example, in a study performed by Ebrahimi et al., the incidence of significant RAS was 31% in patients with HTN [14]. In another study conducted by Rimoldi et al., the prevalence of RAS was 8% after multivariate analysis in hypertensive veterans referred for coronary angiography [15]. In addition, Rihal et al. found that 47.2% of the patients with HTN had RAS, of whom 28% had less than 50% stenosis, and 19.2% had stenosis of 50% or more [16]. However, in our study, the prevalence of RAS in the patients with HTN referred for cardiac catheterization was 28.3%. Consequently, the prevalence of RAS in our study was apparently similar to that reported in the Western nations; whereas, it was higher than that in both Shah et al.’s study performed in Peshawar and a study conducted on Asian populations by Yamashita et al., who found the prevalence of significant RAS as 13% in Japanese patients with HTN [17][18]. This difference may be explained by location and ethnicity. The high prevalence of RAS may be related to the high prevalence of CAD in the Iranian population, especially in Bushehr; furthermore, there is a high prevalence of risk factors for atherosclerosis in both coronary and renal arteries in this country. The risk factors for RAS are those associated with the development of atherosclerosis in other populations. The prevalence of the condition increases with age, a fact first demonstrated in previous studies undertaken almost half a century ago [19]. Moreover, Ozkan et al., found that advanced age and HTN are closely associated with significant RAS and occur more frequently in older individuals [20]. In a recent study, based on color Duplex sonography in 269 patients RAS was present in 11% of the patients in the 50-59 years age group, 18% in the 60-69 years and 23% at the age of 70 years and above [21]. The association between old age and RAS in our study was similar to that described in other researches and more studies have shown that patients with significant RAS are older [22][23][24]. Here, there is a strong and independent relation between old age and RAS which shows a later or slower start of atherosclerosis in renal arteries rather than the coronary or other peripheral vascular atheroscleroses [25]. Although they met the enrollment criteria, we detected RAS infrequently among individuals less than 60 years of age. This observation has important practical implications for cardiac catheterization based RAS screening. The major clinical predictors for RAS are advanced age, multiple coronary artery disease, poorly controlled HTN and renal insufficiency [26]. In our study, a strong relationship between RAS and the number of coronary artery lesions was found. However, this finding was expected considering the high prevalence of RAS in hypertensive patients undergoing angiographic procedures for CAD. De Carvalho found that 74% of patients with RAS had concurrent CAD in one or more coronary arteries, although they had no symptoms of coronary insufficiency [6]. In addition, Ollivier et al. reported that among 650 patients suffering from CAD, 94 presented with RAS, corresponding to an estimated prevalence of 14.5%, including 6% of patients with left coronary artery stenosis, 5.4% with right coronary artery stenosis, and 3.1% with bilateral stenosis [26]. In addition, the results of medical records of consecutive patients who underwent coronary angiography showed that multi vessel CAD was more frequent in patients with significant RAS [23]. However, the relationship between the number of significant coronary lesions and the prevalence of RAS was complex and unclear. Based on angiographic studies, it is commonly accepted that when the number of diseased coronary arteries increases, RAS would probably increase [12][23][27]. In our study, abnormal renal angiographies were found to have no relationship with smoking, gender, BMI, DM, ejection fraction (EF), or HLP. In contrast, some studies showed that there were no significant atherosclerosis risk factors [26][28]. Therefore, it seems that risk factors for the development of atheromatous changes in renal arteries may be different from conventional CAD risk factors, and the detection of risk factors is important because RAS is a progressive disease and may lead to end-stage renal failure.

The high prevalence of RAS in coronary vascular patients with HTN that are undergoing coronary angiography, especially those with 2 VD or 3 VD, shows HTN to be a predictive finding for RAS in HTN patients. On the other hand, according to the prevalence of RAS in developing countries, renal angiography after coronary angiography may be helpful in finding unknown RAS, and could be of assistance in the treatment of patients.
